# Outcomes and complications of prophylactic incisional gastropexy in 766 dogs (2009–2019).

**DOI:** 10.1186/s13104-023-06595-6

**Published:** 2023-10-31

**Authors:** Miranda de la Vega, S. Christopher Ralphs

**Affiliations:** Ocean State Veterinary Specialists, East Greenwich, Rhode Island, USA

**Keywords:** Canine incisional gastropexy, Prophylactic gastropexy, Gastropexy complications, Gastropexy outcomes

## Abstract

**Objective:**

To report the outcomes and complications associated with prophylactic incisional gastropexy performed in dog breeds at risk for GDV.

**Results:**

Seven hundred and sixty-six dogs underwent prophylactic incisional gastropexy of which 61 were electively performed at the time of castration or spay and 705 were adjunctively performed at the time of emergency abdominal surgery. All dogs had short-term follow-up, and 446 dogs (58.2%) had additional follow-up with a median long-term follow-up time of 876 days (range 58-4450). Only 3 dogs (0.4%) had a direct complication associated with the gastropexy site including hemorrhage causing hemoabdomen (2) and infection with partial dehiscence (1). No dogs with long-term follow-up experienced gastric dilatation (GD), gastric dilatation volvulus (GDV), or persistent GI signs following gastropexy. Results of this study found that complications directly associated with prophylactic gastropexy were rare and limited to hemorrhage causing hemoabdomen and infection with partial dehiscence. Transient postoperative GI signs may occur. Gastropexy malpositioning and bowel entrapment were not encountered. There was no occurrence of GD or GDV.

## Introduction

Gastric dilatation volvulus (GDV) is an acute, life-threatening condition affecting a wide variety of dog breeds, most commonly large- and giant-breed dogs. Predisposing factors associated with GDV include increasing age, being underweight, history of GDV in a first-degree relative, rapid eating, once daily feedings, exercising either before or following meals, fearful or anxious temperaments, increased thoracic depth-to-width ratio, and increased hepatogastric ligament length [1–12]. A recent history of splenectomy may be associated with GDV, but reports are conflicting [12, 13].

Mortality rates associated with GDV range from 10 to 55% [9, 14–17], so prophylactic gastropexy has been recommended due to reduced mortality up to thirty times in certain breeds at risk for GDV (e.g. Great Danes) compared with dogs that did not get a gastropexy [19]. Gastropexy is a surgical procedure involving fixation of the pyloric antrum region of the stomach to the right body wall with several techniques described [18–39]. The incidence of gastric dilatation (GD) in patients who previously underwent prophylactic incisional gastropexy (IG) was 11.1% in one report [37]. That study included 27 dogs with postoperative complications of mild and transient GI signs including episodes of regurgitation, inappetence, and gastrointestinal (GI) upset [37]. Gastric malpositioning has also been reported as an intraoperative complication, most recently reported in 5% of dogs that underwent laparoscopically assisted gastropexy (LAIG) [38].

Concerns remain regarding the occurrence of rare but potentially life-threatening complications such as hemorrhage and bowel entrapment [40, 41]. This study aims to report the outcomes and identify the incidence of intraoperative and postoperative complications associated with prophylactic gastropexy in a large population of at risk dog breeds. We hypothesized that performing prophylactic gastropexy in dogs poses minimal risk for intraoperative and postoperative complications.

## Materials and methods

Medical records obtained from Ocean State Veterinary Specialists between 2009 and 2019 were retrospectively evaluated for dogs in which prophylactic incisional gastropexy procedure was performed, including IG, LAIG, and total laparoscopic incisional gastropexy (TLIG). Inclusion criteria were at-risk dog breeds for GDV presenting for elective prophylactic gastropexy or emergency abdominal surgery that also underwent prophylactic IG during laparotomy. Dogs were excluded if they had pre-existing GI disease such as GD, GDV, mesenteric torsion, colonic torsion, and inflammatory bowel disease (IBD) as any postoperative GI signs could not be distinguished from complications of the gastropexy. Dogs were also excluded if they died prior to discharge if cause of death was reported due to the primary disease or a complication from the primary disease (e.g. disseminated intravascular coagulopathy). Medical records were evaluated for signalment, gastropexy technique performed, and intraoperative and postoperative complications.

All dogs were treated with similar anesthetic protocols. Surgical techniques for prophylactic gastropexy included IG by open laparotomy, LAIG using the technique described by Rawlings et al. [28], and TLIG with an endoscopic suturing device (Endostitch; Covidien, Mansfield, MA) using the technique described by Deroy et al [42]. Surgery was performed by either a Diplomate of the American College of Veterinary Surgeons (DACVS), surgery resident, intern, or emergency veterinarian. Any intraoperative complications associated with the gastropexy were recorded. Postoperatively, dogs were managed and discharged based on clinician judgement.

Median follow-up times (with ranges) were reported. The overall follow-up time was defined as the time from surgery until death or last contact. Short-term follow-up involved reviewing records from discharge until incision recheck and/or suture or staple removal, either in-person or through communication with the owner, ranging from 7 days to 2 months to allow for healing of incisions and incision-related complications. Long-term follow-up involved reviewing records beyond incision recheck until death or last contact from the owner or primary care veterinarian.

The frequency of complications as well as the occurrence of GD and GDV following prophylactic gastropexy was reported. Minor complications included any superficial incisional issues that resolved with medical care. Major complications included dogs needing deep incisional and/or abdominal surgery for treatment of a life-threatening postoperative complication such as incision infection and dehiscence, body wall dehiscence, septic abdomen, hemoabdomen, etc. Complications directly associated with the gastropexy found during subsequent abdominal surgery were reported separately as well as any incidence of postoperative GI signs including hyporexia, anorexia, regurgitation, vomiting, and diarrhea and any later abdominal surgeries after discharge.

## Results

Seven hundred and sixty-six (766) dogs met the criteria for inclusion. Sixty-seven different breeds were represented with Labrador Retriever (19.2%, n = 147), Golden Retriever (13.3%, n = 102), and German Shepherd Dog (6.6%, n = 51) being the most common. The sex distribution was 61% male (119 intact, 348 castrated) and 39% female (120 intact, 179 spayed). The median age was 4.9 years (range, 0.2–15.8), and the median weight was 33 kg (range, 6.4–120). Sixty-one dogs presented for elective prophylactic gastropexy at the time of castration or spay (ovariohysterectomy or ovariectomy based on clinician preference), of which there were 19 IG, 41 LAIG, and 1 TLIG. The remaining 705 dogs presented for emergency abdominal surgery where prophylactic IG was also performed. There were 165 unique combinations of abdominal and non-abdominal procedures performed with IG (see Table 1).

**Table 1:** Emergency abdominal procedures performed in addition to IG

Procedure	Number of times procedure performed
gastrotomy	239
enterotomy	194
splenectomy	160
ovariohysterectomy (for pyometra, concurrent mastectomy, or intact status at time of other abdominal procedure)	85
liver biopsy	70
castration	44
enterectomy	35
foreign body milked into the colon	35
serosal patch	13
omental biopsy	10
liver lobectomy	10
dermal mass removal	10
GI biopsies	8
abdominal lymph node biopsies	8
Cesarean section for dystocia	6
cystotomy	6
abdominal and/or inguinal cryptorchidectomy	5
body wall herniorrhaphy	6
mastectomy	4
intestinal tear repair	4
closed suction drain placement	4
dewclaw removal	4
enteroplication	3
cystectomy	3
blepharoplasty	3
prolapsed nictitans gland tacking	2
ovariectomy for ovarian remnant syndrome	2
prostatic cyst/abscess omentalization	2
laparoscopic liver biopsies	2
femoral head and neck ostectomy	2
adrenalectomy	2
nephrectomy	2
negative explore for suspected GIFB	2

Seven hundred and fifty dogs had notation of suture type used for the gastropexy. The suture types used for the gastropexy were polydioxanone (PDS, Ethicon) size 0 (n = 603), 2 − 0 (n = 19), and 3 − 0 (n = 2); poliglecaprone 25 (Monocryl, Ethicon) size 2 − 0 (n = 1); polypropylene (Prolene, Ethicon) size 0 (n = 20) and 2 − 0 (n = 1); barbed glycomer 631 (V-loc, Covidien) size 0 (n = 1) and 2 − 0 (n = 103); or not specified (n = 16). The gastropexy was secured in a continuous (n = 652) or interrupted (n = 8) pattern or not available in the records (n = 106). Orientation and length of gastropexy incisions were inconsistently noted.

All 766 dogs had short-term follow-up with a median of 13 days (range 7–55). Four hundred and forty-six dogs (58.2%) had additional follow-up with a median of 876 days (range 58-4450). Postoperative complications included 88 minor wound-related complications (11.5%) and 22 major complications (2.9%) requiring surgical intervention within the hospitalization or short-term follow-up period. Minor complications included superficial incision infection (84), incision abscess (3), and draining tract (1) that resolved with antibiotic therapy. Only 3 dogs had a direct (major) complication that could be associated with the gastropexy site including hemorrhage from the gastropexy site causing hemoabdomen (2) and infection with partial dehiscence (1). Table 2 lists the other major complications. The frequency of direct gastropexy complication was 0.4%.

**Table 2:** Major complications

Complication	Number of dogs affected
incision infection and body wall dehiscence without septic abdomen requiring revision surgery	8
**hemorrhage from the gastropexy site causing hemoabdomen**	**2**
incision abscess requiring debridement surgery and drain placement	2
superficial incision dehiscence managed by tie-over bandage management until wound closure several days later	2
septic abdomen following enterectomy dehiscence	2
incision infection and body wall dehiscence with septic abdomen requiring revision surgery	1
**incision infection and body wall dehiscence with infection and partial dehiscence of the gastropexy**	**1**
incision and body wall dehiscence without septic abdomen requiring revision surgery that was performed by the primary care veterinarian	1
septic abdomen following enterotomy dehiscence	1
septic abdomen due to ruptured hepatic abscess	1
splenic laceration causing hemoabdomen during laparoscopy portal placement requiring conversion to laparotomy	1

There were 22 major complications including 3 directly associated with the gastropexy

Of the 705 dogs that presented for abdominal emergency surgery and had IG performed, 466 dogs presented for a primary GI emergency and 239 dogs for a non-GI abdominal emergency. 145/466 dogs with a GI emergency had postoperative GI signs (144 presented for GIFB and 1 for intestinal mass resection), and 28/239 dogs with a non-GI abdominal emergency had postoperative GI signs. Table 3 lists the causes identified in the total 173 dogs that had postoperative GI signs. Of note, 11 dogs had transient postoperative GI signs that resolved with medical therapy (variable based on clinician judgment) and definitive diagnosis was not identified with an overall incidence of 1.4%. Three had persistent GI signs that did not respond to medical therapy and did not get additional diagnostic workup with an overall incidence of 0.4%.

**Table 3:** Causes of postoperative GI signs

Cause		Number of dogs affected
GIFB obstruction		106
Gastroenteritis due to dietary indiscretion		34
Unknown etiology		14
	Transient and resolved with medical management (11)	
	Persistent but lost to follow-up (3)	
IBD		7
Adverse reaction to NSAID administration		3
Neoplasia		3
Colonic torsion		1
Hepatopathy		1
Pyelonephritis		1
Megaesophagus		1
omental abscess over an ulcerated enterotomy site		1
Thoracic carcinomatosis		1

A total of 173 dogs had postoperative GI signs reported during follow-up of prophylactic gastropexy

Of the 144 dogs with GIFB obstruction, 93 dogs had subsequent abdominal surgeries, and 80 of those were confirmed to have an intact gastropexy adhesion with proper positioning (range, 15-4450 days after the initial surgery with gastropexy). The remaining 13 dogs did not mention gastropexy in the operative report. None of the 446 dogs with long-term follow-up in this study were diagnosed with GD or GDV. Gastropexy malpositioning and bowel entrapment were not encountered in any dogs that had subsequent abdominal surgery.

## Discussion

The efficacy of prophylactic IG has previously been confirmed for the long-term prevention of GDV in dogs [37]. However, complications associated with IG have not been directly examined. This is the largest retrospective study to date reporting the outcomes and complications of prophylactic IG and identified two major complications including hemorrhage from the gastropexy site leading to hemoabdomen and infection and partial dehiscence of the gastropexy. Prophylactic gastropexy may also cause transient postoperative GI signs, but gastric malpositioning and bowel entrapment were not encountered.

There are 3 recent studies that reported on the long-term outcomes and complications of prophylactic IG in 27 dogs in Benitez et al. [37], LAIG in 44 dogs in Loy-Son et al. [38], and TLIG in 39 dogs in Giaconella et al [39]. In the present study, only 3/766 dogs (0.4%) were found to have complications directly associated with the gastropexy that required surgical intervention compared to a frequency of 0–5% in the other studies evaluating prophylactic IG [37], LAIG [38], and TLIG [39]. Loy-Son et al. was the only study to report the need for immediate surgical revision in 2/44 dogs (5%) due to direct gastropexy complication (gastropexy malpositioning). There was no incidence of gastropexy malpositioning in the present study.

Hemoabdomen from the gastropexy site is possible due to a neurovascular bundle frequently encountered during incision of the transversus abdominis muscle that can lead to significant hemorrhage. This may be a lateral branch of caudal superficial epigastric artery coursing alongside costoabdominalis or iliohypogastricus cranialis nerve (ventral branches of T13 and L1 spinal nerves, respectively) within the transversus abdominal muscle plane [43]. Hemorrhage is usually self-limiting, but electrocautery can be used for hemostasis or to make the gastropexy incisions as originally described by Funkquist in 1967 and 1979. Coagulopathy was considered in both dogs in this study but neither were confirmed.

Two possibilities were considered for the source of gastropexy infection and dehiscence: spread of infection from the ventral midline laparotomy incision through the linea dehiscence to the nearby gastropexy site, or penetration and contamination of suture in the gastric lumen during gastropexy suturing. The latter was considered less likely given that the bacterial loads of the canine stomach are relatively low [44]. Culture of the incision or gastropexy site was not performed. Long-term follow-up was not available for this dog.

Persistence of GI signs and occurrence of GD following gastropexy for GDV in dogs is attributed to underlying gastric motility disorder or negative effects on gastric motility from circumcostal gastropexy [45–47]. More recently, studies by Gazzola et al. and Coleman et al. concluded that LAIG did not influence GI motility based on data collected from a wireless motility device [48, 49]. Three dogs (0.4%) in the present study had persistent GI signs and 11 dogs (1.4%) had transient GI signs that responded to medical therapy compared to 0-7.7% of dogs with GI signs reported in other studies [37–39]. A previous study reported the occurrence of GD in 3/27 dogs (11.1%) after IG [37]. No dogs in the present study with long-term follow-up were diagnosed with GD or GDV.

The overall complication rate in this study was 14.4%. Minor complications (11.5%) only included incision complications as all other complications (major, 2.9%) required additional surgery for revision. Therefore, the surgical site infection rate for all abdominal surgeries in this population of dogs was 11.5%. The frequency of incision complications in this study was similar to the frequency of 5.1–20.4% in other studies evaluating prophylactic IG [37], LAIG [38], and TLIG [39].

This is the largest study to date reporting the outcomes and complications of prophylactic gastropexy. This study concludes that prophylactic gastropexy is a safe procedure that should be performed in at-risk dog breeds at the time of elective castration or spay or as an adjunctive procedure during emergency abdominal surgery. Direct risks associated with prophylactic gastropexy include hemorrhage, infection, and dehiscence. Prophylactic gastropexy may cause transient GI signs that can be medically managed but is unlikely to cause persistent GI signs. The benefits of preventing GDV seemingly far outweigh the low incidence of complications.

## Limitations

- Long-term follow-up was not available for all dogs.
- A prospective study with a large population of healthy at-risk dog breeds without pre-existing GI signs presenting for elective incisional gastropexy at the time of castration or spay would be needed to evaluate the direct effect of gastropexy on causing postoperative GI signs or other complications.

## Data Availability

The datasets used and/or analyzed during the current study are available from the corresponding author on reasonable request.

## Abbreviations

GI: gastrointestinal
GIFB: gastrointestinal foreign body
GD: gastric dilatation
GDV: gastric dilatation volvulus
IBD: inflammatory bowel disease
IG: incisional gastropexy
LAIG: laparoscopically assisted gastropexy
TLIG: total laparoscopic gastropexy
NSAID: non-steroidal anti-inflammatory drug
